# Supplementary material for: Mechanical vibration patterns elicit behavioral transitions and habituation in crawling Drosophila larvae
Source: eLife. 2023 Oct 19;12:e69205. doi: 10.7554/eLife.69205 (PMC10586805; doi:10.7554/eLife.69205)
Supplement: Transparent reporting form [file elife-69205-transrepform1.docx]

***eLife’s* transparent reporting form**

We encourage authors to provide detailed information *within their submission* to facilitate the interpretation and replication of experiments. Authors can upload supporting documentation to indicate the use of appropriate reporting guidelines for health-related research (see [EQUATOR Network](about:blank)), life science research (see the BioSharing Information Resource), or the [ARRIVE guidelines](about:blank) for reporting work involving animal research. Where applicable, authors should refer to any relevant reporting standards documents in this form.

If you have any questions, please consult our Journal Policies and/or contact us: [editorial@elifesciences.org](about:blank).

**Sample-size estimation**

- You should state whether an appropriate sample size was computed when the study was being designed
- You should state the statistical method of sample size computation and any required assumptions
- If no explicit power analysis was used, you should describe how you decided what sample (replicate) size (number) to use

Please outline where this information can be found within the submission (e.g., sections or figure legends), or explain why this information doesn’t apply to your submission:

We did not do an explicit power analysis when designing the study, and have stated this directly in the text along with an explanation of how we arrived at our typical numbers for the number of experiments and number of animals per experiment. This is found in the text of the Materials and Methods section, under the Data Analysis sub-section.

**Replicates**

- You should report how often each experiment was performed
- You should include a definition of biological versus technical replication
- The data obtained should be provided and sufficient information should be provided to indicate the number of independent biological and/or technical replicates
- If you encountered any outliers, you should describe how these were handled
- Criteria for exclusion/inclusion of data should be clearly stated
- High-throughput sequence data should be uploaded before submission, with a private link for reviewers provided (these are available from both GEO and ArrayExpress)

Please outline where this information can be found within the submission (e.g., sections or figure legends), or explain why this information doesn’t apply to your submission:

In all figure captions, we state the number of experiments and number of animals used in each experiment. We did not perform technical replicates, because larvae habituate and this would affect experiments, so new animals were used each time. This is noted in the Methods section in the Drosophila Handling sub-section. We did not exclude any outliers in our data.

**Statistical reporting**

- Statistical analysis methods should be described and justified
- Raw data should be presented in figures whenever informative to do so (typically when N per group is less than 10)
- For each experiment, you should identify the statistical tests used, exact values of N, definitions of center, methods of multiple test correction, and dispersion and precision measures (e.g., mean, median, SD, SEM, confidence intervals; and, for the major substantive results, a measure of effect size (e.g., Pearson's r, Cohen's d)
- Report exact p-values wherever possible alongside the summary statistics and 95% confidence intervals. These should be reported for all key questions and not only when the p-value is less than 0.05.

Please outline where this information can be found within the submission (e.g., sections or figure legends), or explain why this information doesn’t apply to your submission:

Statistical methods are described in the Data Analysis sub-section of the Materials and Methods section. We do not have any N<10 data, so raw data is not shown, except for the example trajectory in Fig. 1. The statistical tests used, values of N, SEM, and P-value designations are all in the figure captions. The exact N is not always known, but this does not alter any significance tests or results, and this is noted and explained in the Data Analysis sub-section. Figure 6 is the only place where p-values were computed. In the figure itself we use * or ** to denote p-value ranges, and the exact values are reported in the the Data Analysis sub-section to avoid overly cluttering the caption.

(For large datasets, or papers with a very large number of statistical tests, you may upload a single table file with tests, Ns, etc., with reference to sections in the manuscript.)

**Group allocation**

- Indicate how samples were allocated into experimental groups (in the case of clinical studies, please specify allocation to treatment method); if randomization was used, please also state if restricted randomization was applied
- Indicate if masking was used during group allocation, data collection and/or data analysis

Please outline where this information can be found within the submission (e.g., sections or figure legends), or explain why this information doesn’t apply to your submission:

N/A for this research I think? Larvae of the same age are essentially indistinguishable and were selected randomly from their food plates (this is noted in the Drosophila Handling sub-section).

**Additional data files (“source data”)**

- We encourage you to upload relevant additional data files, such as numerical data that are represented as a graph in a figure, or as a summary table
- Where provided, these should be in the most useful format, and they can be uploaded as “Source data” files linked to a main figure or table
- Include model definition files including the full list of parameters used
- Include code used for data analysis (e.g., R, MatLab)
- Avoid stating that data files are “available upon request”

Please indicate the figures or tables for which source data files have been provided:

Two scripts are provided.

One Matlab script (Larva_Vibration_Universal.m) that combines with previously published data extraction software (which is cited in the Methods section) to extract the behavior considered in all Figures.

One Igor Pro script (DESandRESfits.ipf) that finds uncertainties in fits for desensitization and re-sensitization time constants and computes significance tests. These are used for the analysis shown in Fig. 6.
